# Supplementary material for: Are carbon emissions trading and green financial instruments synergistic? -Comprehensive quantitative research based on content analysis
Source: PLoS One. 2024 Mar 7;19(3):e0298601. doi: 10.1371/journal.pone.0298601 (PMC10919601; doi:10.1371/journal.pone.0298601)
Supplement: S1 Data — (ZIP) [file pone.0298601.s001.zip › Data/地级市Z_F工作报告.zip!/╜¡╬≈/╞╝╧τ╩╨/└┤╫╘╣1⁄2╓┌║┼í╛┬φ┐╦╩2╛▌═°í┐_2017.docx]

各位代表：

现在，我代表区人民政府向大会报告工作，请予审议，并请各位政协委员和列席会议的同志提出意见和建议。

一、2016年工作回顾

过去的一年，在市委、市政府和区委的正确领导下，在区人大、区政协的有力支持下，全区上下认真贯彻落实党的十八大和十八届三中、四中、五中、六中全会精神，按照市委“年年有变化，三年大变样，五年新跨越”的总体要求，围绕“主抓项目、强攻工业，确保实现五个大变样，打造两地三区，让湘东人民过得更好”的总体思路，积极应对各种困难和挑战,把握机遇,奋勇争先,实现了“十三五”各项工作的良好开局。全区完成生产总值194.66亿元，增长8.6%；财政总收入完成18.78亿元，增长3.63%；全社会固定资产投资完成235.24亿元，增长13.2%；社会消费品零售总额完成48.47亿元，增长11.4%；城镇居民人均可支配收入达30856元，增长7.9%；农村居民人均可支配收入达15481元，增长8.5%。一年来的工作主要体现在以下六个方面：

（一）工业经济转型加速。完成规模以上工业增加值86.51亿元，增长8.2%；荣获全省加快工业发展加速工业崛起年度贡献奖、全省第二届最佳优化民营经济发展环境县(市、区)。工业平台日臻完善，湘东产业园西扩片区控制性详规完成初步成果，西区工业大道实现泥沙路通车，一期渡口、横溪、龙形湾三个平台正在推进；工业南大道改建段竣工通车、延伸段泥沙路通车；园区工业供水系统升级工程全面启动；天然气湘东分输站建设获省发改委批复核准；产业园铁路专用线即将启动运营。产业发展势头良好，聘请山东工陶院编制完成了《湘东工业陶瓷产业技术创新中长期发展规划》，并与其签订战略合作协议。全国工业陶瓷产业知名品牌示范区创建加快推进；龙发实业化工陶瓷应用技术工程研究中心获得省工程研究中心（工程实验室）认定批复；华星化工、湘东石化填料被认定为国家级高新技术企业。时代工艺被评为省级服务业龙头企业和省级工业设计中心。金刚科技陶瓷辊棒生产线建成投产，岳峰高分子预计今年5月试产，弘源煤焦化启动复产前期工作。

（二）农业农村加快发展。完成农业增加值18.72亿元，增长3.8%。新增市级以上农业龙头企业4家，新认证“三品一标”3家。天涯种业进入全国种子企业50强，成为全省首个杂交水稻育、繁、推一体化国家级A证企业，科技创新能力跃居国内先进、省内领先。七彩生态农业、锦旺农林获评省级休闲农业示范点；仙居农业、一统林农等6家企业获得省级林业龙头企业认定。连续获得省级生猪调出大县奖励。农村土地确权工作在全省率先完成，是2016年全省唯一完成验收的县区。新农村建设稳步推进，投资6537万元，打造新农村建设点70个，完成主干道和入户路硬化61.2公里，改水2974户，改厕2265户。

（三）城乡面貌日新月异。麻山生态新区建设扎实推进，完成了3.82平方公里启动区控制性详规和麻山河景观方案专项规划设计方案编制；陈家湾路建成通车，陈家湾湿地公园和小桥安置区一期竣工；S533桐田至中环路段启动设计施工；五河治理防洪工程、外环路一期等项目正在抓紧建设。城乡品位不断提升，对城区主干道进行了绿化提升；完成一河两岸三桥及城区亮化；完成城区八条主干道沥青路及人行道升级改造，全面实现弱电入地、雨污分离；G60沪昆挂线改建工程全面贯通；云程公园二期正在建设；启动了萍钢“灯动分开”工程。城乡环境明显改善，建成6座乡镇垃圾压缩中转站，启动城区及9个乡镇污水处理厂建设，环卫市场化工作即将运行；干净湘东、文明创建、国省道整治和打击两违等专项整治深入推进。划定生态保护红线，水泥机立窑生产线全部停产，新建了空气自动监测站。完成造林面积15543.1亩，森林覆盖率达69.84%。

（四）三产服务逐步升温。完成第三产业增加值62亿元，经济增长贡献率提高14.5个百分点。麓林湖养生公馆一期建成，中帜汽配城全面复工，云程望族开盘销售。乡村旅游日趋红火，麻山幸福村入选中国乡村旅游模范村和省生态文明示范基地；锦旺农业生态园被认定为全省森林食品基地。新开发10条乡村旅游精品徒步路线，评选出十佳乡村旅游示范村。乡村旅游扶贫示范区基础设施建设项目获国家发改委审核通过，有望获批专项建设基金1.27亿元。2016年，全区接待旅游人数同比增长31.21%；旅游综合收入同比增长28.68%。

（五）争资引项成效显著。中央预算内专项资金到位9.78亿元；累计争取专项建设基金项目34个，获批项目资本金18.02亿元，列省、市前列。麻山生态新区人居环境建设PPP项目进入财政部第三批示范项目库，成为中国政企合作投资基金（即PPP基金公司）在全国县区首个正式签约合作的PPP项目，获3亿元直接投资。区级融资平台昌盛公司主体信用评级由AA-调升至AA级。招商引资成绩斐然，引进全国500强新希望集团与樟丰化工合作投资4.5亿元新建年产7.5万吨氢氧化钾生产线项目、康裕科技投资3亿元的环保设备及化工填料项目；江能神雾投资62.5亿元的超超临界发电示范项目纳入国家“十三五”能源规划。

（六）民计民生持续改善。围绕改善民生这一核心，全面推进社会事业发展，重点实施了“三院一校一场一路”建设。精准扶贫扎实推进，全年脱贫472户、1280人，广寒官溪村成功脱贫；完成棚户区改造1285户。教育事业蓬勃发展，完成广寒寨中学等11所乡镇中小学标准化建设，湘东中学和湘东职业中专改扩建扎实推进，峡山口、东桥、排上公办中心幼儿园启动建设；一次性解决全区待聘教师聘用问题。卫生计生服务不断优化，区妇幼保健院开工改扩建，东桥卫生院主体建筑基本完成，腊市卫生院提升改造工程即将完工；在全市率先与市人民医院探索组建医联体；城市公立医院综合改革工作全面启动，计划生育服务水平不断提升。社会保障水平有新提升，湘东综合福利中心和区救助站竣工，10所乡镇敬老院改造提升工程全面启动。市场改造步伐加快，春风市场投入使用，河洲市场改造正在实施，乡镇集贸市场改造加快推进。交通、水利等基础设施不断完善，省道S533流田至桐田一级公路升级改造工程基本实现土路通车，S308东桥至界头段改造竣工通车，建设农村公路230公里、桥梁7座，湘麻公路（大江边至景星）改造正在进行招投标前期工作，石甲坊至官桥公路已纳入2017年PSL农村公路项目库第三批进行申报，新垅至和平公路建设正在实施；麻山取水口上移工程竣工通水，源湴防洪工程、大沙江水库项目启动实施。文化事业不断进步，麻山赖氏传统傩面具雕刻公司列为省非物质文化遗产生产性保护示范基地；湘东皮影戏列入省级非物质文化遗产项目名录。村（社区）干部打卡工资由每人每月500元提高到1800元。双拥工作持续进步，荣获全省双拥模范区七连冠。社会保持和谐稳定，获得全省社会治安状况优秀县（市、区）、全省信访工作三无县区称号。安全生产保持平稳态势，完成15家煤矿的引导关闭退出工作，退出产能78万吨，高危行业产业布局逐步优化，获全省安全生产优秀县区。

此外，妇女儿童、民族宗教、人防、老龄、残联、物价、公共机构节能、档案、邮政、通讯、广播电视、石油、烟草、粮食、供销等各项事业都取得了新成绩。人民武装、消防、法治、地质矿产、交警、防灾减灾等各项工作也取得了新进展。在加快经济社会发展的同时，我们不断加强政府自身建设，依法履行审计、监察监督职能，坚持重大事项集体决策，自觉接受人大和政协监督，办理人大代表议案、建议和政协委员提案209件，办复率100%，满意理解率达96%。

成绩的取得，是市委、市政府和区委正确领导的结果，是区人大和区政协监督、支持的结果，更是全区人民齐心协力、奋力拼搏的结果。在此，我代表区人民政府，向人大代表、政协委员和全区人民，向离退休老同志、各民主党派、工商联、无党派人士、人民团体、驻区部队官兵、政法干警，向所有关心、支持和参与湘东发展的各界人士，表示衷心的感谢并致以崇高的敬意！

各位代表，成绩来之不易，历程催人奋进，回首2016年工作，我们觉得：

坚强有力的党的领导是事业发展的前提。过去一年尤其是换届以来，我们坚定不移按照中央、省、市的决策部署，在区委的坚强领导下，紧紧围绕区十一次党代会确定的总体思路，以“开局就是决战、起步就是冲刺”的信念和决心，展现出前所未有的良好精神风貌，全区经济社会发展取得了明显的进步，赢得了鼓舞人心的成绩。实践证明，只有坚定不移地坚持党的领导，我们的各项事业发展才会加快，突破各种阻碍的能力才会增强，人民群众的获得感就会更丰富。

　　团结协作的干群队伍是干好工作的保证。过去湘东一直被戏称为“灰姑娘”，为改变城乡环境卫生“脏、乱、差”的现状，全区干部群众戮力同心，一同上阵，掀起了干净湘东、文明创建“大会战”，湘东的“颜值”迅速提升，取得了超乎寻常的进步，在全市两月一次的城市管理考评中连续夺得第一的好成绩，极大地鼓舞了干劲、激发了斗志，形成了团结干事的浓厚氛围。实践证明，团结就是最大的力量，只要上下同向，干群同心，就会形成干事创业的强大合力。

　　实干担当的工作作风是实现目标的关键。过去一年，全区各条战线上的同志不分昼夜，加班加点，付出了艰辛的努力，确保了全区各项重点工作扎实推进，湘东也在全市项目观摩中取得了第二名的好成绩，迅速地塑新了湘东的形象。实践证明，天上不会掉馅饼，担当才会有所作为，实干才能梦想成真。唯有作风“换挡”，干劲“提速”，才能确保各项工作的高效落实。

永不服输的拼劲韧劲是赶超跨越的动力。一年来，我们始终抱着“不达目的不罢休”的决心，自我加压，敢于超越，努力让不可能成为可能，变后进为先进。市政府工作报告已明确市级工业平台落户湘东，城乡环境大改善，取水口上移等重点工程如期完成，等等，无不体现出了湘东干群不甘人后、勇于争先的奋斗精神。实践证明，只有保持“咬定青山不放松”的韧劲和永不服输的拼劲，才能突破困境，克服难题，不断开创发展新局面。

同时，我们也清醒地认识到，全区经济社会发展还面临不少困难和问题，主要有：全区经济总量和质量还不高，经济增速仍处低位运行，财政总收入与预期目标还有一定差距；企业创新步伐不快，转型发展任务艰巨；区内实有税源不足，刚性支出与日俱增，财政收支平衡压力日益加大；城乡环境综合治理需持续用功，城市建设和管理有待加强；社会事业发展与群众期盼还有差距，环境保护、安全生产、社会稳定等方面工作依然存在较大压力；等等。这些困难和问题，亟待我们在加快发展中努力解决。

二、2017年工作安排

2017年，全区经济社会发展的指导思想是：全面贯彻党的十八大和十八届三中、四中、五中、六中全会以及中央、省、市经济工作会议精神，坚持“稳中求进，奋发求为”的工作总基调，围绕“年年有变化、三年大变样、五年新跨越”的总体要求，始终把握“主抓项目、强攻工业，确保实现五个大变样，打造两地三区，让湘东人民过得更好”这一总体思路，积极适应和把握经济发展新常态，着力提升发展质量和效益，统筹抓好稳增长、促改革、调结构、惠民生、防风险等各项工作，切实推动全区经济社会发展行稳致远，以优异成绩迎接党的十九大胜利召开。

2017年，全区经济社会发展的主要预期目标是：生产总值增长 8%左右，财政总收入增长5%左右，全社会固定资产投资增长 13%左右，规模以上工业增加值增长8%左右；社会消费品零售总额增长10 %左右，城镇和农村居民人均可支配收入分别增长8 %和8.5 %左右，城镇登记失业率控制在4.5 %以内。

为实现上述目标，我们将在以下六个方面下功夫：

（一）坚定不移强攻工业，不断提升工业竞争力

湘东发展的希望在工业，工业发展的希望在质量和效益的提升。要顺应供给侧结构性改革的发展要求，坚定不移推动工业产业优化结构、转型升级。

1.大力推进工业平台建设。将湘东产业园建成区、西扩区整体规划为大型工业平台，加强与经济开发区的对接合作，启动湘东飞地工业园建设，着力打造全市工业主阵地。做好湘东产业园区申报《中国开发区公告目录》工作，争取更多的政策支撑。按照“规模化、标准化、园林化”的建设理念，加快推进湘东产业园和园区西扩平台建设，确保西区工业大道、工业南大道延伸段、日供15000吨工业供水系统扩建工程和工业污水处理厂等基础设施全面竣工，天然气湘东分输站项目启动建设，渡口片区、横溪片区和龙兴湾片区工业平台初步成型，平整土地2000亩以上，新建标准化厂房2万平方米以上。狠抓物流平台建设，积极争取市铁路货运站西迁至姚家洲火车站，盘活萍乡电厂、萍乡铝厂等闲置土地；开通湘东产业园铁路专用线，打造集仓储、铁路联运于一体的区域物流中心。

2.大力加快产业集群发展。围绕打造“3+1”产业，制定出台工业产业集群发展规划，加快发展工业陶瓷、彩印包装、精细化工三大产业集群，积极引进和培育战略性新兴产业集群。以金刚科技、龙发实业、普天高科、东陶陶瓷等企业为纽带，加强与工业陶瓷南北两院（佛山陶瓷研究所、山东工陶院）的对接合作，推动传统陶瓷产品向全国一流的非金属新材料及陶瓷成套设备方向发展；围绕打造长江以南最大的钾碱生产基地，推动精细化工产业向集约化、规模化、规范化发展；以时代包装、联合印铁、华雅印务等为载体，加强与湖南工大、江西财大等院校对接合作，促进彩印包装与文化、科技融合，推动传统彩印包装向文化创意设计转型。争取投资62.5亿元的江能神雾超超临界发电项目尽快落地，带动相关衍生产业加快发展。把龙头企业作为培育产业集群的关键，全面落实中央鼓励企业兼并重组的扶持政策，从政策、资金、要素配置等方面倾斜龙头企业，在各产业集群重点扶强做大1-2家科技含量高、发展潜力大的龙头企业，进而带动其他企业加快转型，提升产业集群发展质量和效益。落实《湘东区鼓励企业上市和挂牌优惠政策规定》，帮助万相科技、金刚科技、启才陶瓷等初步达成挂牌意向的企业做好股改挂牌工作，争取年内至少有1家企业在“新三板”挂牌。

　　　3.大力优化工业发展环境。开展优化经济发展环境年活动，坚持“主抓项目、强攻工业”战略，大力营造“重工业、抓工业、兴工业”浓厚氛围，将客商、企业的满意度作为最终评判标准，扎扎实实为客商和企业办实事、解难事，把“爱企、亲商、便民”真正体现在行动上，对破坏工业发展环境的典型案例予以严厉查办，对强揽工程、阻碍生产或施工、寻衅滋事、强买强卖等违法犯罪活动予以重拳出击，严厉查处损害发展环境的违纪违规行为，保持打击涉企违法犯罪高压态势。对重大产业项目及招商项目、企业反映的问题，不遮掩、不回避、不拖沓，坚持在一线指挥、一线服务、一线解决问题，让客商和企业进得来、留得住、发展好。

　　 （二）坚定不移主抓项目，不断提升发展支撑力

项目是纲，纲举目张。项目工作的成效决定湘东今天的和未来的发展希望，要牢固树立“抓项目就是抓发展”理念，坚定不移推动各个领域项目建设。

1.高起点谋划落实项目。紧跟国家政策调整方向，确保中央预算内项目资金到位8亿元以上，力争9亿元；继续拼抢专项建设基金、政策性银行扩张贷款、PSL、亚行贷款等项目投资发展机遇，精心包装一批事关全局和长远发展的重大项目，争取进入国家、省、市项目库，为实现“年年有变化、三年大变样、五年新跨越”获得更多资本，奠定坚实的基础。

2.高质量招商引进项目。完善招商引资考核激励机制，明确乡镇年度招商任务，调整入园项目和发展总部经济税收分配方式，最大限度让利给招商主体乡镇，做到既有压力也有动力。严格招商准入门槛，围绕产业发展规划，积极对接中材高新等央企、上市公司，引进一批“高科技、高投入、高产出、高附加值、强带动力”的“四高一强”项目，着力培育经济发展“新引擎”。

3.高要求加快建设项目。坚持项目挂点、定期调度等好做法，组织实施好专项建设基金项目，加快推进岳峰高分子、中节能垃圾焚烧发电、金刚科技二期等在建项目尽快投产达标，启动樟丰化工第二条生产线建设，有效提高项目的落户开工率、投产达标率和财税贡献率。

4.高效率盘活提升项目。积极推进基础较好的传统产业复产提升，为企业牵线搭桥，帮助企业靠大联强，克服技术、融资等方面瓶颈，加快转型升级步伐；重点扶持弘源煤化工等企业早日复产，帮助龙发实业、普天高科、石化填料、启才陶瓷、东陶陶瓷等企业加快转型提升步伐，进行产业延链、补链、强链，使传统企业焕发新生机。

（三）坚定不移发展农业，不断提升农旅融合力

湘东农村要发展，农民要致富，农旅结合是出路。要发挥好特色农业和生态资源优势，以“一区一品一带多节庆”为抓手，坚定不移推动农业供给侧结构性改革和农旅融合发展。

1.打造现代农业示范区。推进农业标准化建设，不断完善农业基础设施，注重产业融合发展，积极拓展“农业+”模式。以加快省级现代农业示范园区麻山核心园区建设为引领，着力打造以锦旺农林为核心的腊市现代农业示范园、以一统、丰瑞等油茶公司为主体的省级油料种植加工示范园，以及全省绿色生猪养殖示范基地和山羊养殖示范基地；同时，加快益农社建设，以电子商务为载体发展订单农业，提升农业供给和销售能力，力争通过1-2年的努力成功创建国家级现代农业示范区。

2.打造农业知名品牌。争创国家种子基地县，推动天涯种业进行股份制改造，争取3年内实现IPO（即主板上市），早日进入全国种子企业前5强；鼓励争创省级、国家级农业产业化龙头企业，积极推介七彩葡萄新工艺酿酒（不含二氧化硫）和“赣西两头乌”等地方特色品牌，培育一批有自主品牌的农产品，力争2017年全区新增农产品“三品一标”认证4家以上。

3.打造百里特色农业休闲观光带。按照农旅结合、连片发展的思路，在319国道、麻凤线、陈广线沿线着力打造以麻山、腊市、排上、东桥、广寒、白竺为核心，农业、生态、旅游、文化等要素融合发展的百里特色农业休闲旅游观光带，集中连片打造一批果蔬、养殖、休闲观光等特色基地，着力打造“一镇一景”“一村一品”。抓好麻山幸福村国家4A级景区和排上仙居山庄、麻山双月湾湿地公园全省4A级乡村旅游点创建申报工作；积极推介麻山生态新区、老关凯丰纪念馆、荷尧彭公景秀等养生文化游、红色文化游、宗教文化游；抓好“油菜花节”“龙舟赛”等节庆活动。

4.打造美丽乡村样板。按照全省新农村建设行动规划要求，加快编制区域总体规划，采取填空补穴的方式，在陈广线、麻凤线、320国道沿线成线成片打造新农村示范点，确保每个乡镇打造1个新农村建设示范点（其中4个为区乡共建点），从今年开始，力争通过四年的努力，实现全区新农村建设“扫一遍”，建设“整洁美丽、和谐宜居”新农村。

（四）坚定不移优化环境，不断提升湘东软实力

改善城乡面貌是湘东人民长期以来的期盼，要进一步提升湘东形象，必须坚定不移推动城乡环境大改善、大变样。

1.持续加大城区建设力度。推进老城区扩容提质，启动文体新区建设。做好城市详规与市“四规合一”对接工作，围绕提升老城区对市级工业平台的配套承接功能，对老城区扩容提质进行科学规划，将昌盛大桥以西片区纳入城区扩容区域，把文化会展中心、青少年活动中心、学校、医院、体育馆、滨河公园、龙舟码头、景观坝、星级酒店等城市功能要素纳入规划；加快《萍乡市湘东城区东部片区控制性详细规划》、《萍乡市湘东区文化体育新区概念规划》编制进度，启动城区综合管线、停车场、公共交通等专项规划编制，同步推进征拆和招商工作。加快城区基础设施建设改造提升。推进城区水、电、气、路网络改造提升，完成萍钢“灯动分开”工程；完成城区所有主干道沥青路面铺设和小街小巷改造亮化工程，确保昌盛大道改造6月份完工，启动27条城区小街小巷沥青路面铺设工程；全面启动文体新区、新街升级改造和云程公园二期建设、老城区五河治理防洪工程；启动文化体育中心、图书馆、美术馆等文化场馆建设，在主城区启动1所区级二甲医院建设，完成区妇幼保健院改扩建工程。

2.持续加快特色小镇建设。结合地域特色和文化，按照差异化、产业化、特色化的思路，着力打造一批产业发展“有特色”、功能叠加“能聚集”、制度供给“显活力”的特色小镇。加快完善麻山新区基础设施，推进“萍水十景”、外环路、S533延伸线、佳沙洲大桥、桐田安置小区等先行重点项目建设，完成河道治理、桐田桥新建、善洲桥修复等项目，并以麓林湖养生公馆、市康复中心为龙头，着力发展养生养老、医疗、健康产业，将麻山生态新区打造成健康特色小镇。依托优美的山水资源和豆腐产业基础，将白竺大丰打造成山水豆腐小镇，挖掘豆腐文化，不断丰富豆腐品种，融合桐花谷等山水旅游资源，发展豆腐产业，形成一户一品、集中连片的特色产业格局。逐步推进以工业陶瓷的产业基础和特色底蕴为支撑的下埠工业陶瓷文化小镇、以东桥生态旅游资源为支撑的赣湘边际旅游小镇建设。

3.持续推进城乡环境治理。坚持干净湘东和文明创建行动常态化，不断激发群众积极性、提高群众参与度，持续推进四清五治一整洁、国省道整治、打击两违等专项整治。完善城乡环卫一体化体系，实施城区环卫市场化，推进垃圾压缩中转站建设乡镇全覆盖，全面实现生活垃圾无害化处理。完善城区及全区村镇规划，出台《湘东城区私房改造规划管理实施意见》，坚决杜绝违章违规建筑；抓好萍水河湘东段河道疏浚，完善城区污水收集管网，建设河洲景观坝，打造“城中湖”，净化湘东河道水质，美化河道景观。加强城乡管理综合执法队伍建设，不断推动城乡建设和管理合理化、规范化，着力开展城区文明创建示范道路、示范市场、示范社区等评比，不断提升湘东对外形象，加快实现城乡环境大变样。

4.持续加强生态环境治理。推进生态文明试验区建设，切实加强源头治理，深入推进“净空、净水、净土”行动。净空方面，推进工业烟粉尘治理，力争年内淘汰一段式煤气发生炉。建立健全“三治”监测网络，加大治超治限治尘力度；购置除尘喷雾车，降低城区粉尘浓度，提高空气质量。净水方面，全面落实“河（库）长制”管理和考核，加强饮用水水源地保护和重点河流水污染治理，加快城乡污水处理设施建设，启动草水流域综合治理PSL项目。净土方面，倡导造林护林意识，继续实行封山育林，严防各类毁林破坏现象；加大对重点区域和企业排污监管，加强土壤污染源头综合治理，让湘东青山常在、绿水长流。

（五）坚定不移改革发展，不断提升创新驱动力

新常态、新经济时代的发展是改革发展动力的较量，是创新驱动能力的较量。加快湘东发展，必须坚定不移推进改革创新，不断增添发展新动能。

1.科技创新引领发展。深入实施创新驱动发展战略，加强与山东工陶院、武汉理工大学等科研院校的对接，争取国家工业陶瓷工程技术研究中心萍乡分中心2017年落户湘东，抓好国家化工陶瓷应用工程技术研究中心筹建工作，不断提升产业配套服务水平；抓好市长质量奖和区长质量奖的落实，支持鼓励企业加大自主创新和科研合作开发，力争年内有2-3项新材料、节能环保领域科技成果转化为现实生产力，工业陶瓷企业产品发明专利达10件以上。

2.积极改革促进发展。主动顺应供给侧结构性改革的发展要求，进一步落实财税体制改革，依托综合治税平台加强区内实有税源的征收管理。进一步深化推进土地管理改革，采取长期租赁、租让并举、先租后让的方式推进工业用地改革，严格工业用地管理，建立低效利用土地市场化退出机制，探索存量土地盘活、土地产出效益与新增建设用地计划指标分配挂钩机制。综合运用市场机制、政策手段，推进产业园区“僵尸企业”转型发展，对长期闲置土地实行“腾笼换鸟”。

3.优化服务助推发展。大力推进项目审批预审制和联审联批制度改革，做到审批事项能减则减、审批程序应简尽简。深化政银企合作，坚持金融债权联席会议制度，持续改善诚信金融环境；进一步完善商业银行考评体系，激励银行系统为全区供给侧结构性改革提供更优金融服务；积极引进各类金融机构入驻，提升全区金融保障水平。

（六）坚定不移改善民生，不断提升社会向心力

民生是人民幸福之基、社会和谐之本。让湘东人民过得更好是我们矢志前行的目标和方向，压力再大也要坚定不移推动民生改善，促稳定、保和谐。

1.实施扶贫工程。按照精准扶贫、精准脱贫方略，实施产业扶贫，推进“一村一品”特色产业、光伏发电、乡村旅游和电商扶贫，增加困难群众收入水平；实施交通扶贫，加快贫困村道路、水利等基础设施建设，改善贫困村生产生活条件；实施就业扶贫，开展职业技能培训，让贫困群众有一技之长；实施健康和教育扶贫，完善医疗、保险等救助体系，改善因病致贫，落实贫困家庭学生助学政策，促进教育脱贫，确保全区5个贫困村、765户贫困户、2044名贫困人口全部实现脱贫。

2.实施惠民工程。实施民生建设“八个全覆盖”，继续推进“三院一校一场一路”建设，持续改善民生福祉。加快公办中心幼儿园建设，确保全区每个乡镇最少有1所公办中心幼儿园。完成腊市、下埠卫生院改扩建项目和东桥中心卫生院一期建设，启动荷尧、老关等乡镇卫生院改扩建和村级卫生计生服务室改造提升。实施敬老院标准化建设和管理提升工程，重点加快腊市、麻山、荷尧、下埠、老关敬老院的建设提升；探索分片区集中供养模式，打造1所全省一流的区域中心敬老院；积极引进社会资本参与养老事业。完成荷尧中学、排上中心小学等13所学校标准化建设；完成湘东中学校园一期整体改造，启动二期校园文化景观建设；完成湘东职业中专扩建主体工程；启动麻山中学新建工程，实施麻山生态新区教育园区建设；争取以优秀等次通过国家义务教育均衡发展督导评估；推进群体事业发展，广泛开展全民健身活动，推广健康生活方式。做好砚田市场重建招商工作，力争河洲市场10月份正式运营；加快推进乡镇集贸市场改造，逐步消除以路为市现象。加大投入力度，完善城乡交通网络，确保S533流田至桐田公路升级改造工程10月份通车，S308荷尧至长平公路改建工程11月份竣工通车；启动工业南大道至小洞塘段、油塘至凤凰、崇源至源湴等重点道路建设。扩展城乡供水一体化覆盖面，抓好冷潭湾水厂改扩建工程和湘东镇、腊市镇、下埠镇管网延伸工程。

3.实施平安工程。实施精准安全监管，坚决防范和遏制较大事故的发生，力争年内关停4万吨以下煤矿，努力确保工矿商贸领域“零事故、零死亡”。加大交通安全投入，完善城乡交通安全设施，加强交通秩序管理，增强群众交通安全意识，着力降低交通事故风险。加强食品药品安全监管力度，建设食品检验检测中心，启动病死畜禽无害化处理厂和生猪屠宰场项目建设。不断完善社会防控体系，推进“天网”“地网”建设，加强巡防队伍规范化建设；深入推进综治网格化管理，扎实做好重点领域、重点群体、重点行业的信访维稳工作，确保社会和谐稳定。

三、加强政府自身建设

各位代表，新的形势、新的目标对政府工作提出了新的要求。政府及政府系统各部门必须强化宗旨意识，忠实履行职责，加强人民政府、法治政府、担当政府、效能政府、廉洁政府“五个政府”建设，不断提升政府的行政能力和服务水平。

（一）加强人民政府建设。全心全意为人民服务是政府的天职，人民政府必须以人民为中心。坚持向人民群众承诺的事，说一项做一项，项项兑现，对人民群众关心的事，定一件办一件，件件算数。在加快发展中，更多地关注民计民生，努力让人民享有更多的发展红利。坚决不搞急功近利的“形象工程”和劳民伤财的“政绩工程”，确保今天和今后我们所做的一切经得起实践、历史、人民的检验。

（二）加强法治政府建设。依法行政是政府工作的基本准则。坚持把政府工作纳入法治化轨道，严格按照法定程序、法定权限、法定职责行使人民赋予的权利，让行政权力授予有据、行使有规、监管有效，促进社会公平正义。紧紧依靠区委领导，自觉接受人大、政协监督，高度重视新闻舆论和人民群众监督，加强政府内部层级监督，保障和支持审计、监察等部门依法独立行使监督权。

（三）加强担当政府建设。重任在肩惟担当。政府必须义不容辞地担当起发展一方经济、富裕一方群众、维护一方平安的使命和责任，大力弘扬锲而不舍的拼搏精神，敢闯敢试的攻坚勇气，雷厉风行的实干作风，做到敢担当、能担当、善担当，以干部的“辛苦指数”换取百姓的“幸福指数”。建立容错纠错机制，为实干者撑腰，为担当者担当，为负责者负责，让想干事的人有机会，能干事的人有平台，干成事的人有地位。

（四）加强效能政府建设。强化“注重结果、追求效果、多出成果”的理念，弘扬“八小时内抓紧干、八小时外多奉献”的奉献精神，构建勤政、快捷、高效的执行体系。坚决纠正懒政、怠政行为，严厉查处失职、失责行为。健全考核奖惩机制，按进度比绩效，按实绩论英雄，激发工作干劲，努力形成干部带头干、带着干的良好局面。

（五）加强廉洁政府建设。清正是为官之德，廉洁是民心所向。认真履行政府系统党风廉政建设主体责任和“一岗双责”，不断加强工程建设、土地出让、政府采购等重点领域的制度建设和专项治理，规范从政行为，构建“不敢腐、不能腐、不想腐”的廉政环境。秉承政府过“紧日子”，群众和企业过“好日子”的理念，以廉洁的形象取信于民。

各位代表，湘东是我们的共同家园，人民的信任永远是我们前进的动力。让我们在市委、市政府和区委的坚强领导下，以蓬勃向上、克难奋进的精神状态，以只争朝夕、全力跨越的豪迈干劲，锐意进取，真抓实干，为让湘东人民过得更好而努力奋斗，以优异的成绩迎接党的十九大胜利召开！
